# First report of *Yokenella regensburgei* isolated from external auditory canal after diving in valley

**DOI:** 10.1002/ccr3.5177

**Published:** 2021-12-13

**Authors:** Gina Na, Je Eun Song, Jeonghyun Chang

**Affiliations:** ^1^ Department of Otolaryngology‐Head and Neck Surgery Inje University, Ilsan Paik Hospital Goyang Korea; ^2^ Department of Internal Medicine Inje University, Ilsan Paik Hospital Goyang Korea; ^3^ Department of Laboratory Medicine Inje University, Ilsan Paik Hospital Goyang Korea

**Keywords:** 16S rRNA sequencing, otitis externa, pathogen, *Yokenella regensburgei*

## Abstract

*Yokenella regensburgei* is a Gram‐negative, oxidase‐negative motile rod which is rarely isolated from human caused a few opportunistic infections in immunocompromised patients so far. We report the first case of otitis media combined with externa caused by *Y*. *regensburgei* in an immunocompetent patient. A 56‐year‐old male patient visited the outpatient clinic of the Otolaryngology Department due to otorrhea of the right ear started after diving in mountain valley in Korea 3 days ago. He was immunocompetent adult and clinical examination revealed swelling and debris accumulation in the right external auditory canal with an intact, erythematous tympanic membrane, and clear, odorless otorrhea. Microbiological culture of otorrhea revealed *Y*. *regensburgei* by matrix‐assisted laser desorption/ionization time‐of‐flight and PCR‐based 16S rRNA gene sequencing. His otorrhea persisted, and a pinpoint perforation occurred in the inferior anterior portion of the tympanic membrane. 50% acetic acid irrigation and 500 mg of oral ciprofloxacin were prescribed, and his infection was cured after 4 weeks. This is the first case of otitis media combined with externa caused by *Yokenella regensburgei* in an immunocompetent patient. Given that *Yokenella* species infections are rare, especially in immunocompetent patients, this case highlights the importance of history taking and communication between clinicians and laboratory physicians. Molecular identification methods assist in identifying rare pathogens.

## Graphical abstract

We report the first case of otitis media combined with externa caused by *Yokenella regensburgei* in an immunocompetent patient. This case highlights the importance of history taking and communication between clinicians and laboratory physicians.

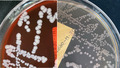

## 1 INTRODUCTION

*Yokenella regensburgei* is a Gram‐negative, oxidase‐negative motile rod first described by Kosako et al. in 1984.^1^ It belongs to the family Enterobacteriaceae and is the only species of the genus *Yokenella*. *Y*. *regensburgei* is rarely isolated from humans and its clinical significance is unclear. It typically causes opportunistic infections in immunocompromised patients.^2^, ^3^ We report the first case of otitis media combined with externa caused by *Y*. *regensburgei* in an immunocompetent patient.

## 2 CASE REPORT

**FIGURE 1:**
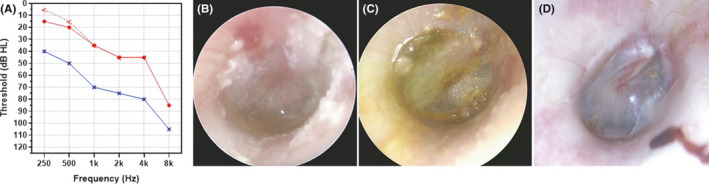
(A) Pure‐tone audiogram showing conductive hearing loss at low frequencies (250 and 500 Hz) in the right ear (red). (B) Right external auditory canal swelling (not shown) and an inflamed eardrum were detected when the patient visited the outpatient clinic. Whitish debris and clear otorrhea were noted. (C) After 50% acetic irrigation for 1 week, otorrhea persisted, and tympanic perforation developed. (D) After taking ciprofloxacin for 2 weeks, the tympanic perforation was healed, and the otorrhea disappeared

**FIGURE 2:**
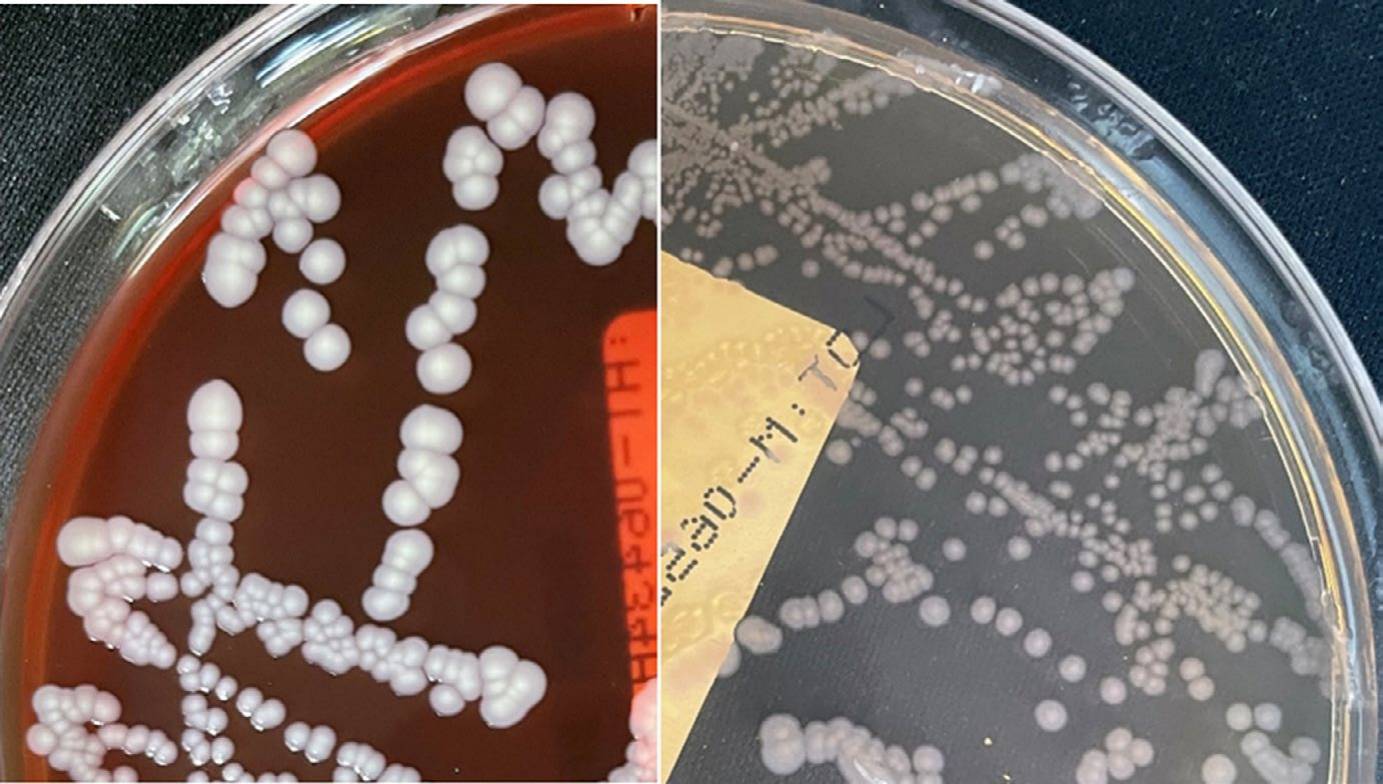
*Y*. *regensburgei* colonies on sheep blood agar (left) and MacConkey agar (right)

A 56‐year‐old male patient visited the outpatient clinic of the Otolaryngology Department due to otorrhea of the right ear. It had started 3 days prior during diving in Gapyeong Valley, a mountainous area of South Korea. He experienced mild pain as his right ear touched the water surface while diving, but did not complain of subjective hearing loss, dizziness, or fever. However, he felt water in the ear canal. Clinical examination revealed swelling and debris accumulation in the right external auditory canal (EAC) with an intact, erythematous tympanic membrane (TM) and clear, odorless otorrhea (Figure 1B). In addition to the pre‐existing hearing loss, a pure‐tone audiogram showed 10 and 5 dB air‐bone gaps at 250 and 500 Hz, respectively (Figure 1A). He was taking aspirin and clopidogrel for hypertension and previous myocardial infarction. Microbiological culture was performed on swab specimens for otorrhea. Specimens were transferred to a clinical microbiology laboratory and inoculated on chocolate agar and 5% sheep blood agar. Gram staining showed frequent white blood cells and Gram‐negative rods. Small grayish colonies had grown on MAC and BAP agar with grade of ‘many’ after 1 day (Figure 2). Identification by matrix‐assisted laser desorption/ionization time‐of‐flight (MALDI‐ToF) mass spectrometry using a Vitek MS (bioMérieux, Marcy‐L'Etoile, France) revealed *Yokenella regensburgei*. PCR‐based 16S rRNA gene sequencing identified the isolate as *Y*. *regensburgei*, revealing 99.72% identity (1403/1407 bp) with *Y*. *regensburgei* ATCC 49455(T) (GenBank accession number JMPS01000045). Susceptibility testing was performed using the Vitek 2 Automated Antimicrobial Susceptibility System (bioMérieux). The isolate was susceptible to amikacin, cefotaxime, ceftazidime, cefepime, ciprofloxacin, gentamicin, ertapenem, imipenem, tigecycline, piperacillin/tazobactam, trimethoprim/sulfamethoxazole, and aztreonam, and resistant to ampicillin, cefazolin, and amoxicillin/clavulanic acid. Before pathogen identification, debridement and 50% diluted acetic acid irrigation were applied. During week 2, EAC swelling and otalgia had improved but otorrhea persisted, and a pinpoint perforation occurred in the inferior anterior portion of the TM (Figure 1C). Because of middle‐ear infection due to diving‐related tympanic injury, 500 mg of oral ciprofloxacin was prescribed, twice daily for 2 weeks. During week 3, otorrhea was recultured and *Y*. *regensburgei* was no longer isolated. The TM perforation was completely healed by week 4, and the EAC had dried up (Figure 1D). The patient's ear infection and hearing loss were then completely treated.

## 3 DISCUSSION

We describe a case of otitis media combined with otitis externa caused by *Y*. *regensburgei* in a healthy immunocompetent adult. *Y*. *regensburgei* is a member of the bacterial flora of insects and has been recovered from their intestinal tract.^2^ It rarely infects humans, although a few cases of opportunistic infection of immunocompromised hosts have been reported—septicemia in HIV patients, brain abscess in lymphoma patients, necrotizing fasciitis in an immunocompromised host, and diabetic foot in a patient with chronic kidney disease who had undergone kidney transplantation.^3^ A case of infectious diarrhea with sepsis was reported in an immunocompetent male in 2017 in Greece.^4^ This is the second case of *Y*. *regensburgei* infection in a healthy adult.

At the first visit, the TM was intact after EAC cleansing, and history taking suggested otitis externa, which is common worldwide, particularly in swimmers.^5^ History taking revealed that the patient was infected with *Y*. *regensburgei* during swimming in a mountain valley. To date, there has been no report of otitis externa caused by *Y*. *regensburgei*. To our knowledge, this is the first case report of *Y*. *regensburgei* otitis media with otitis externa.

Topical rather than oral antibiotics are recommended in uncomplicated otitis externa due to locally high concentrations.^6^ However, in this case, TM perforation suggested intercurrent otitis media. Also, given that the ear canal was narrow and anteriorly overhanging, ear drying was likely to be insufficient. Therefore, an oral agent was applied alone rather than in combination with topical antibiotics. Antimicrobial susceptibility testing of the isolate revealed resistance to ampicillin, amoxicillin/clavulanic acid, and cefazolin. This is largely in agreement with previous reports of resistance to at least two of ampicillin, cefazolin, and amoxicillin/clavulanic acid.^2^, ^3^, ^7^, ^8^, ^9^

It is not possible to accurately identify *Y*. *regensburgei* using the automated identification systems typically available in clinical microbiology laboratories. *Y*. *regensburgei* biochemically resembles *Hafnia alvei* and can be misidentified as such by routine identification methods.^2^, ^3^ MALDI‐ToF and 16S rRNA sequencing can be used for species‐level identification. MALDI‐ToF identification is inexpensive and easy, so is recommended for difficult‐to‐identify pathogens. In addition, providing clinicians with information on rare pathogens will improve patient management.

## 4 CONCLUSION

We report the first case of otitis media combined with externa caused by *Yokenella regensburgei* in an immunocompetent patient. Given that *Yokenella* species infections are rare, especially in immunocompetent patients, this case highlights the importance of history taking and communication between clinicians and laboratory physicians. Molecular identification methods assist in identifying rare pathogens.

## CONFLICTS OF INTEREST

No conflicts of interest.

## AUTHOR CONTRIBUTIONS

G.N involved in history taking and manuscript draft writing. J.E.S. involved in editing and manuscript review. J.C involved in manuscript editing.

## CONSENT

Written informed consent was obtained from the patient to publish this report in accordance with the journal's patient consent policy.

## Data Availability

Research data are not shared.
